# Localization of phosphotyrosine adaptor protein ShcD/SHC4 in the adult rat central nervous system

**DOI:** 10.1186/s12868-019-0541-5

**Published:** 2019-12-10

**Authors:** Hannah N. Robeson, Hayley R. Lau, Laura A. New, Jasmin Lalonde, John N. Armstrong, Nina Jones

**Affiliations:** Department of Molecular and Cellular Biology, University of Guelph, Guelph, ON Canada

**Keywords:** ShcD, Shc4, ShcB, ShcC, Neural progenitor cells, Olfactory bulb

## Abstract

**Background:**

Mammalian Shc (Src homology and collagen) proteins comprise a family of four phosphotyrosine adaptor molecules which exhibit varied spatiotemporal expression and signaling functions. ShcD is the most recently discovered homologue and it is highly expressed in the developing central nervous system (CNS) and adult brain. Presently however, its localization within specific cell types of mature neural structures has yet to be characterized.

**Results:**

In the current study, we examine the expression profile of ShcD in the adult rat CNS using immunohistochemistry, and compare with those of the neuronally enriched ShcB and ShcC proteins. ShcD shows relatively widespread distribution in the adult brain and spinal cord, with prominent levels of staining throughout the olfactory bulb, as well as in sub-structures of the cerebellum and hippocampus, including the subgranular zone. Co-localization studies confirm the expression of ShcD in mature neurons and progenitor cells. ShcD immunoreactivity is primarily localized to axons and somata, consistent with the function of ShcD as a cytoplasmic adaptor. Regional differences in expression are observed among neural Shc proteins, with ShcC predominating in the hippocampus, cerebellum, and some fiber tracts. Interestingly, ShcD is uniquely expressed in the olfactory nerve layer and in glomeruli of the main olfactory bulb.

**Conclusions:**

Together our findings suggest that ShcD may provide a distinct signaling contribution within the olfactory system, and that overlapping expression of ShcD with other Shc proteins may allow compensatory functions in the brain.

## Background

The Shc (Src homology and collagen) family of adaptor molecules plays an important role in mediating signaling events that link activated cell surface proteins such as receptor tyrosine kinases (RTKs) to intracellular signaling pathways [1]. Shc proteins are characterized by the presence of an amino-terminal phosphotyrosine binding (PTB) domain, a central collagen homology 1 (CH1) region which contains a series of phosphorylatable tyrosine residues, and a carboxy-terminal Src homology 2 (SH2) domain. The interaction capabilities of these domains position Shc proteins within a variety of signaling complexes that regulate processes such as cellular differentiation, proliferation, and migration [1, 2].

The four identified members of the Shc family are ShcA/Shc/*SHC1* [3], ShcB/Sli/*SHC2* [4], ShcC/Rai/N-Shc/*SHC3* [4–6], and the most recently discovered and least-characterized homologue, ShcD/*SHC4* or RaLP (Rai-like protein) [7, 8]. As a result of alternative initiation codon usage and differential splicing, multiple isoforms exist for ShcA and ShcC [3, 6]. ShcD is most similar to p66ShcA, and both possess an amino-terminal CH2 domain unique to the longer Shc isoforms [8]. ShcD deviates from the other Shc proteins with an additional 3 tyrosine residues in the CH1 region and loss of the central adaptin binding motif, which impacts trafficking of the EGFR [9].

In addition to sequence and structure divergence, members of the Shc family differ in their spatiotemporal expression. During brain development, ShcA is found within dividing neural stem/progenitor cells (NPCs), though this expression declines over time such that at maturity, it is primarily expressed outside of the central nervous system (CNS) [6, 10]. In contrast, ShcB and ShcC are largely restricted to the CNS, and expressed in the mature adult brain [4–6, 11], with ShcC gradually replacing ShcA as NPCs progress towards a postmitotic phenotype [12]. While much less is known about ShcD expression, it has been detected in multiple sub-regions of the adult mouse brain [8], in skin and melanocytes [7], and in the neuromuscular junction where it signals with the MuSK RTK [8]. In the developing mouse embryo, ShcD is present throughout the CNS, as well as in skeletal and cardiac muscle, epithelia of several organs, and multiple neural crest-derived tissues [13]. Despite the prominent expression of ShcD in the CNS, its precise distribution and cellular localization therein has yet to be determined. In this report, we have used immunohistochemistry and double staining approaches to examine the pattern of ShcD expression in the adult rat brain and spinal cord, and compared this profile with those of the neuronally enriched ShcB and ShcC proteins.

## Results

### Cellular distribution of ShcD in the adult rat brain

**Fig. 1:**
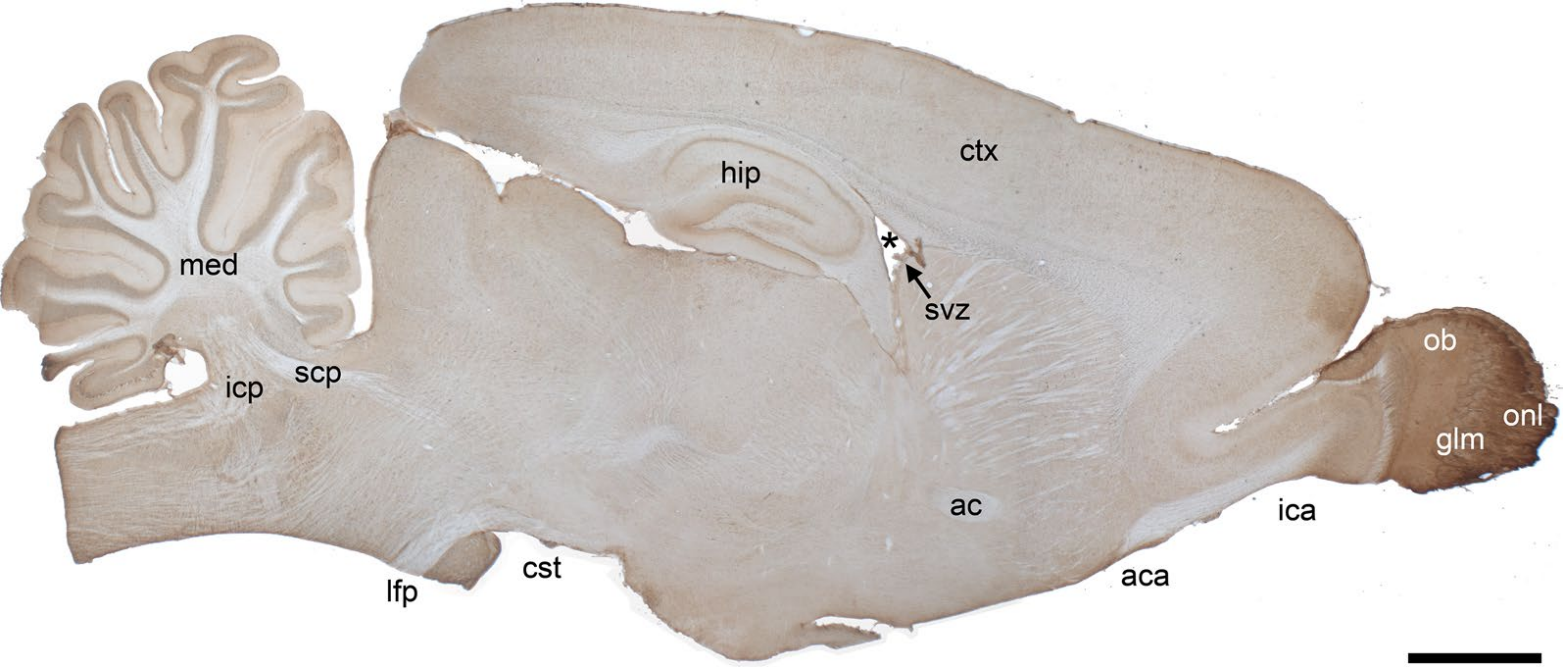
Immunolocalization of ShcD in the adult rat brain. ShcD is broadly distributed throughout the brain, with intense staining in the olfactory bulb (ob), as well as regions of the hippocampus (hip) and cerebellum (med). Parasagittal section of an 8-week old male Sprague–Dawley rat is shown. No staining was apparent when the primary antibody was omitted from the reaction. Superior and inferior cerebellar peduncle (scp, icp), corticospinal tract (cst), longitudinal fasciculus of the pons (lfp), lateral ventricle (denoted by *), subventricular zone (svz; arrow), cortex (ctx), intrabulbar (ica) and anterior limb (aca) of the anterior commissure (ac), olfactory nerve layer (onl), glomerular layer (glm). Scale bar = 2 mm

To examine the neural localization of Shc proteins, sections were prepared from adult rat brain and stained using ShcB, ShcC or ShcD-specific antibodies which we have previously validated for immunohistochemistry [13]. ShcD distribution in the mature brain appears relatively widespread, with somata and dendrites of most principal cells displaying ShcD immunoreactivity (Fig. 1). ShcD staining is most prominent within the olfactory nerve layer, where axons of the olfactory sensory neurons travel en route from the nasal mucosa to the olfactory bulb before synapsing in the glomeruli. Increased expression is also detected in specific subregions of the cerebellum and hippocampus, as well as in the subventricular zone (SVZ). By contrast, ShcD staining is rather diffuse in axons of many fiber tracts. The immunostaining patterns observed for ShcB and ShcC were highly similar to those reported previously on rat brain tissue [11, 12] and are compared in detail with ShcD below.

### Cellular distribution of ShcD compared with ShcB and ShcC in the olfactory bulb

**Fig. 2:**
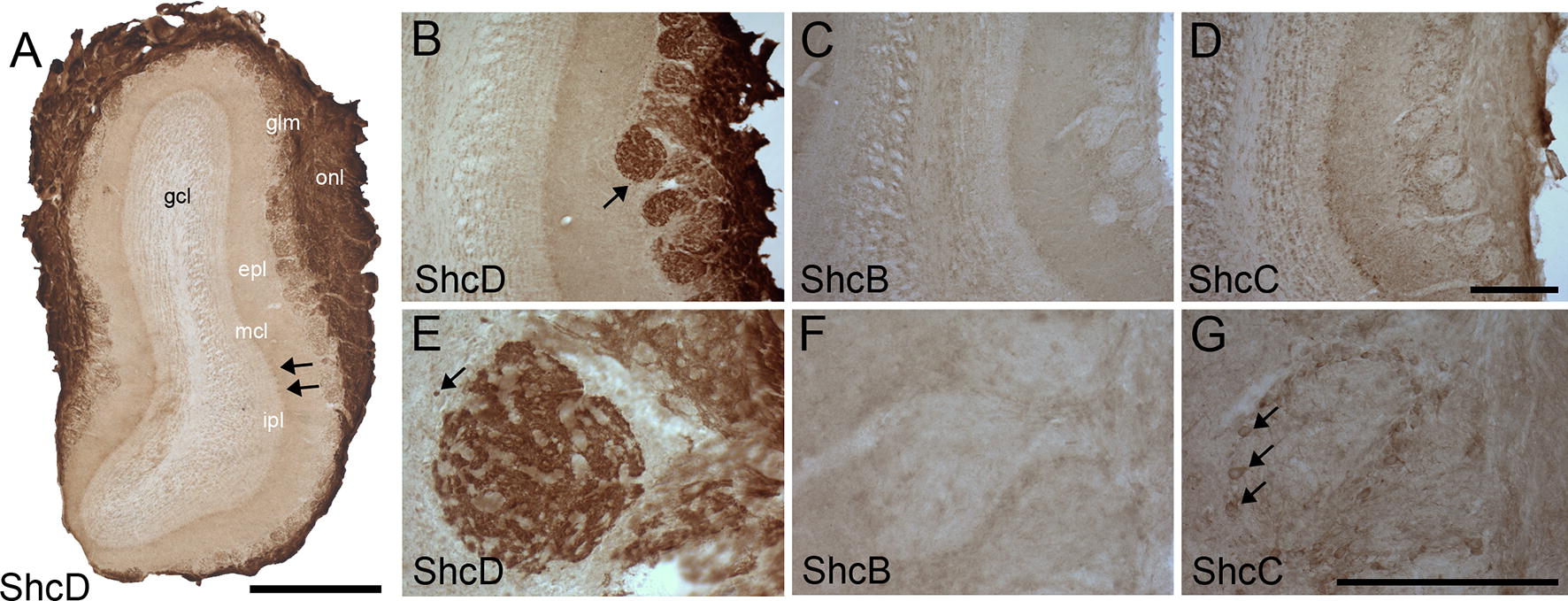
Distribution of ShcD compared with ShcB and ShcC in the rat olfactory bulb. Low-power (**A**) and high power (**B**, **E**) photomicrographs of transverse (coronal) sections show intense ShcD immunoreactive axons and terminals in the glomeruli (glm) and olfactory nerve layer (onl). ShcD positive staining is also detected within the granule cell layer (gcl), mitral cell layer (mcl; see arrows in **A**), and in tufted cells of the external plexiform layer (epl; see arrows in **B** and **E**). Little or no ShcB (**C**, **F**) or ShcC (**D**, **G**) staining is apparent in olfactory sensory neurons, though weak signal for ShcC can be detected in the somata and dendrites of interneurons located in the periglomerular region (see arrows in **G**). inner plexiform layer (ipl). Scale bars: **A** = 2 mm, **B**–**D** = 200 μm, **E**–**G** = 80 μm

We next profiled expression of the three neural Shc proteins in specific anterior to posterior structures. ShcD shows the highest immunoreactivity in axons and terminals of the glomerular and olfactory nerve layers (Fig. 2A, B and E). It can also be seen within all other layers of the olfactory bulb, including in distinct cell bodies of the mitral cell layer and central granule cell layer, and in external tufted cells between the external plexiform layer and the glomerular layer (Fig. 2A, B and E). Faint diffuse staining of ShcB (Fig. 2C, F) is present throughout the olfactory bulb, although no specific immunoreactivity is found in the glomeruli or olfactory nerve layer. ShcC can be observed in the somata and dendrites of interneurons located in the granule cell layer (Fig. 2D) and periglomerular region (Fig. 2G), as well as in mitral cells (Fig. 2D).

### Cellular distribution of ShcD compared with ShcB and ShcC in the cerebral cortex, hippocampus and dentate gyrus

**Fig. 3:**
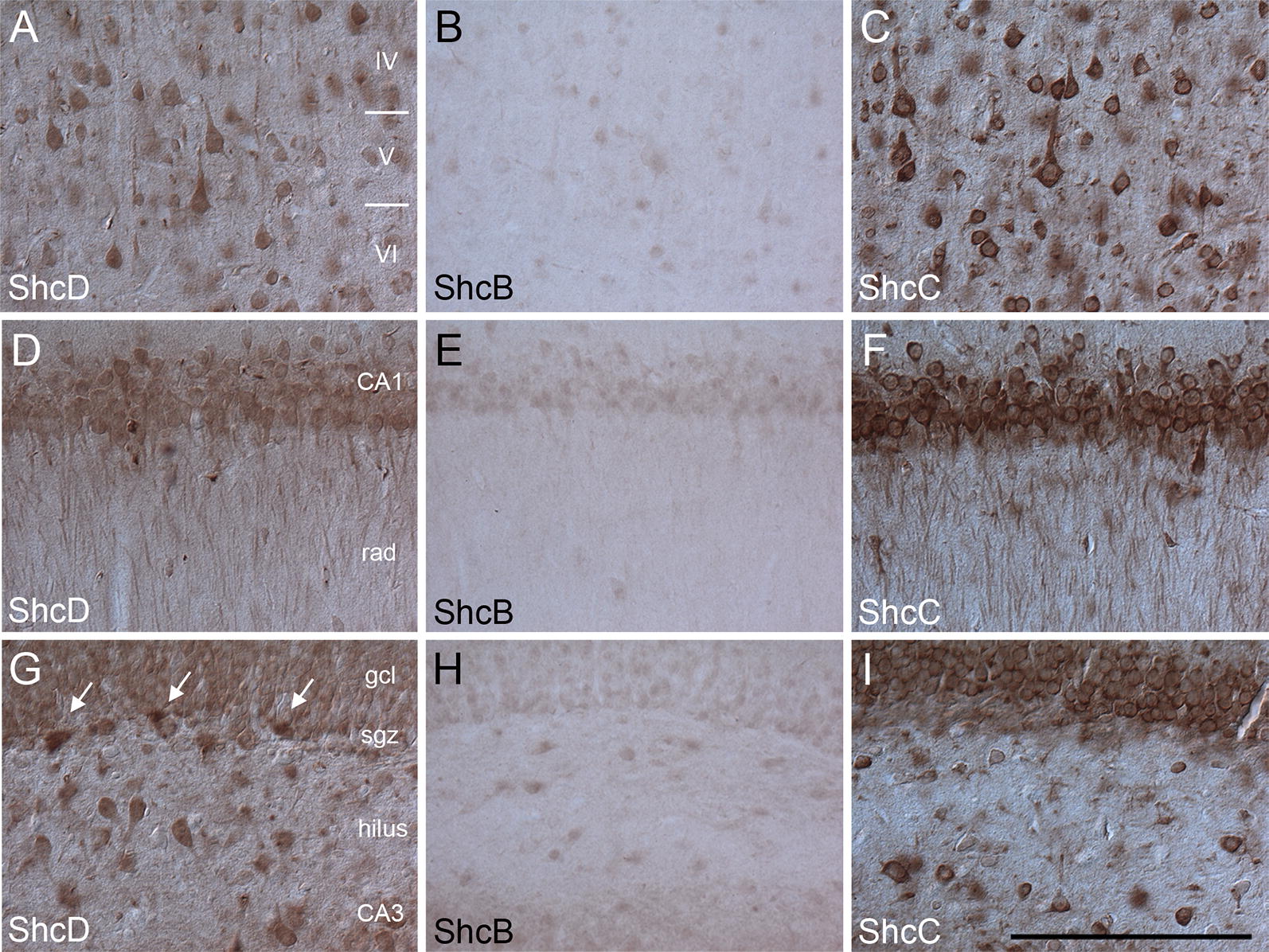
Immunoreactivity of ShcD compared with ShcB and ShcC in the rat neocortex and hippocampus. ShcD (**A**, **D**, **G**), ShcB (**B**, **E**, **H**) and ShcC (**C**, **F**, **I**) staining is present in the somata and dendrites of pyramidal cells in the neocortex (see layers IV, V and VI; **A**–**C**) and the hippocampus (CA1, CA3, stratum radiatum (rad); **D**–**F**) as well as granule cells (gcl) and neurons of the hilus of the dentate gyrus (**G**–**I**). Note the intense staining of ShcD compared to ShcB and ShcC in distinct cells within the subgranular zone (sgz) (**G**–**I**; see arrows in **G**). Scale bar: **A**–**I** = 200 μm

Within the neocortex and hippocampus, Shc proteins display different intensities of staining, with ShcC appearing most darkly stained in these regions, followed closely by ShcD, with only faint staining for ShcB. In the neocortex, ShcB, ShcC and ShcD immunoreactivity is present within somata and dendrites of the large pyramidal neurons in layer V (internal pyramidal layer or ganglion cell layer) (Fig. 3A–C). Staining is prominent in the cytoplasm of these cells, consistent with the role of Shc proteins as intracellular adaptors. In the hippocampus proper, Shc staining can be detected in pyramidal cells of CA1 and CA3 areas as well as in fibers of the stratum radiatum (Fig. 3D–F). In the dentate gyrus, both ShcC and ShcD (but not ShcB) are readily detected in the granule cell layer (Fig. 3G–I); however, ShcD appears to be more intensely stained in the sparsely scattered neurons within the hilus, and even further enriched in distinct cells comprising the subgranular zone (SGZ) (Fig. 3G).

### Cellular distribution of ShcD compared with ShcB and ShcC in the cerebellum

**Fig. 4:**
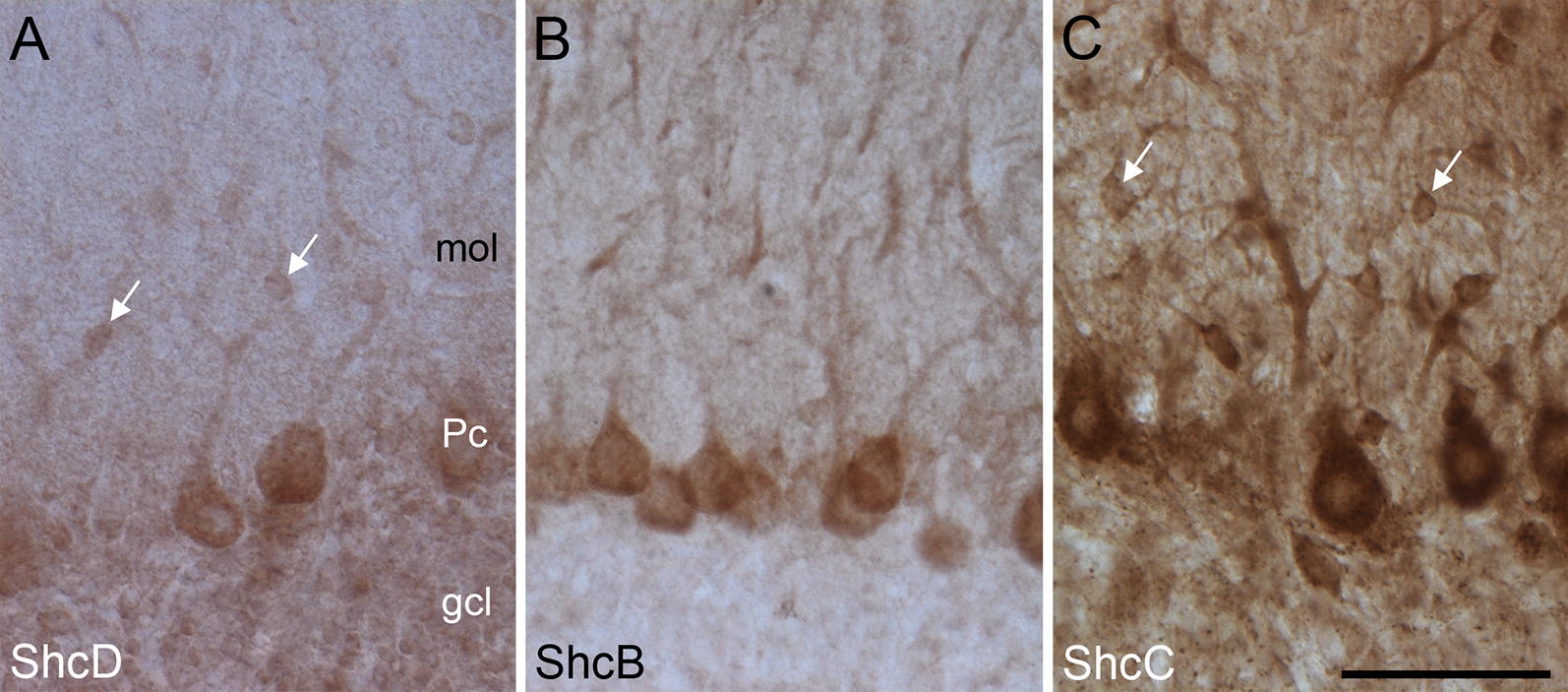
Distribution of ShcD compared with ShcB and ShcC in rat cerebellar neurons. **A** Granule cell layer (gcl), Purkinje cells (Pc) and interneurons (arrows) in the molecular layer (mol) of the cerebellum display diffuse ShcD immunoreactivity. **B** Only Purkinje cell somata and dendrites are immunoreactive for ShcB. **C** ShcC staining is darkest and it is prominent throughout all three layers of the cerebellum. Arrows denote interneurons. Scale bars: **A**–**C** = 50 μm

In the cerebellar cortex, all Shc proteins are present, though they exhibit distinct staining patterns across the three layers. ShcD immunoreactivity is weak but detectable within the inner granule cell layer, the central Purkinje cell layer and interneurons of the outer molecular layer (Fig. 4A). ShcC immunoreactivity is similarly apparent throughout all three layers of the cerebellum, though the staining is much darker than that of ShcD, particularly in Purkinje cell somata, apical dendrites and associated spines (Fig. 4C). By contrast, only Purkinje cell somata and dendrites show ShcB staining (Fig. 4B).

### Cellular distribution of ShcD compared with ShcB and ShcC in the brainstem

**Fig. 5:**
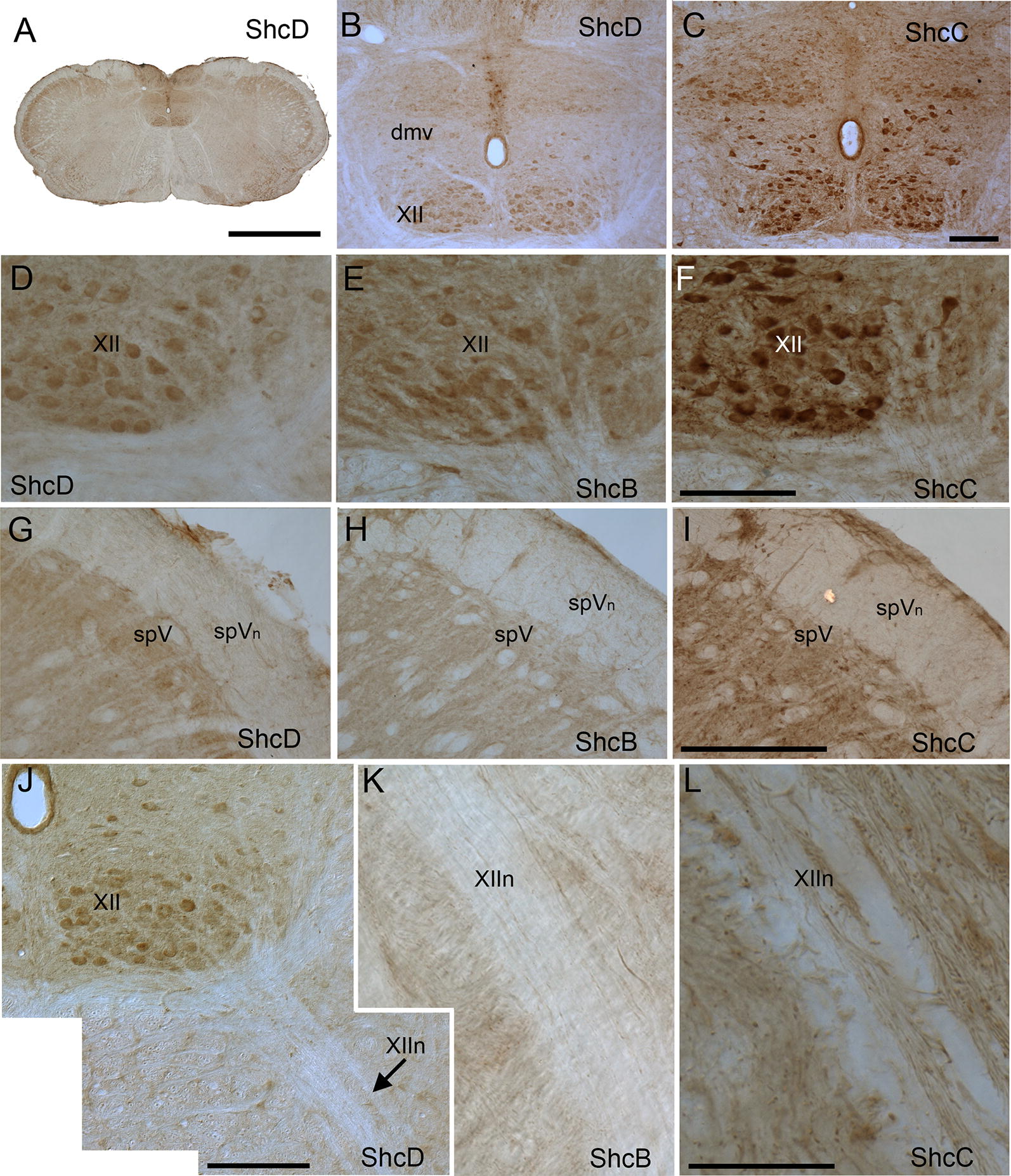
Localization of ShcD compared with ShcB and ShcC in the rat brainstem. Low-power (**A**) and high-power (**B**–**L**) photomicrographs of ShcD (**A**, **B**, **D**, **G**, **J**), ShcB (**E**, **H**, **K**) and ShcC (**C**, **F**, **I**, **L**) immunoreactivity in transverse sections through the level of the hypoglossal nuclei (XII) and nerve (XIIn). Motor neurons stain positive for all Shc proteins, while fiber tracts are immunonegative for ShcD. Dorsal motor nucleus of the vagus nerve (dmv), spinal trigeminal nerve (spVn), spinal nucleus of the trigeminal nerve (spV). Scale bars: **A** = 1 mm, **B**, **C** = 200 μm, **D**–**F** = 200 μm, **G**–**I** = 100 μm, **J** = 200 μm, **K**, **L** = 100 μm

In the brainstem, differential staining of Shc proteins can again be observed, with intense ShcC reactivity, as well as robust levels of ShcD which are comparable to ShcB (Fig. 5A–C). Immunoreactivity localized within neuronal somata and dendrites can be discerned, particularly in the clusters of large motor neurons in the hypoglossal nucleus and dorsal motor nucleus of the vagus (Fig. 5A–F). Interestingly, axons and fiber tracts appear to be relatively immunonegative for ShcD compared to ShcC, including the spinal trigeminal nerve (Fig. 5G–I) and the axons of the hypoglossal motor neurons exiting as the hypoglossal nerve (Fig. 5J–L).

### Cellular distribution of ShcD compared with ShcB and ShcC in the spinal cord

**Fig. 6:**
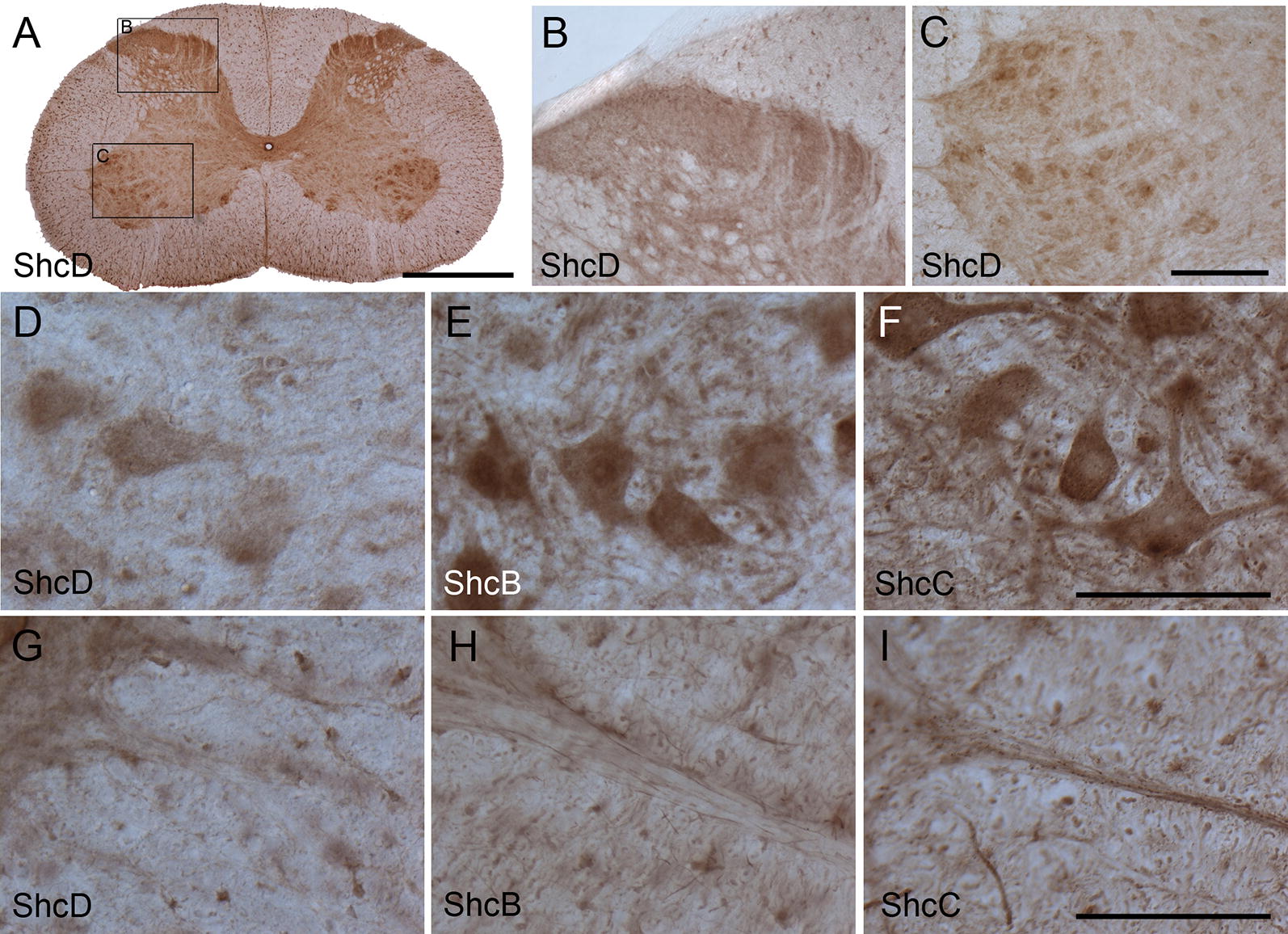
Immunoreactivity of ShcD compared with ShcB and ShcC in the rat spinal cord. ShcD is found throughout the spinal cord (**A**), with prominent staining in axons and cell bodies of neurons in the dorsal (**B**) and ventral (**C**) horns. All Shc proteins are detected in ventral motor neurons (**D**–**F**), though ShcD labeling is weakest in these cells and their surrounding neuropil (**D**). Immunoreactivity for ShcB (**H**) and ShcC (**I**) but not ShcD (**G**) is seen in ventral motor nerves. Scale bars: **A** = 1 mm, **B**, **C** = 200 μm, **D**–**F** = 100 μm, **G**–**I** = 100 μm

Within the spinal cord, ShcD distribution appeared widespread (Fig. 6A), with clear staining in axons and cell bodies of sensory neurons in the dorsal horn (Fig. 6B), motor neurons in the ventral horn (Fig. 6C), as well as in the neuropil. However, ShcD displayed the weakest labeling intensity of all the Shc proteins in the spinal cord. ShcD staining of the ventral motor neurons was diffuse compared to the intense ShcB and ShcC labeling of ventral motor neurons and surrounding neuropil (Fig. 6D–F). Similarly, both ShcB and ShcC but not ShcD could be observed in the ventral motor nerves (Fig. 6G–I). High ShcD immunoreactivity is present within the axon tracts within the white matter (Fig. 6A).

### Cellular distribution of ShcD compared with ShcB and ShcC in the trigeminal ganglion

**Fig. 7:**
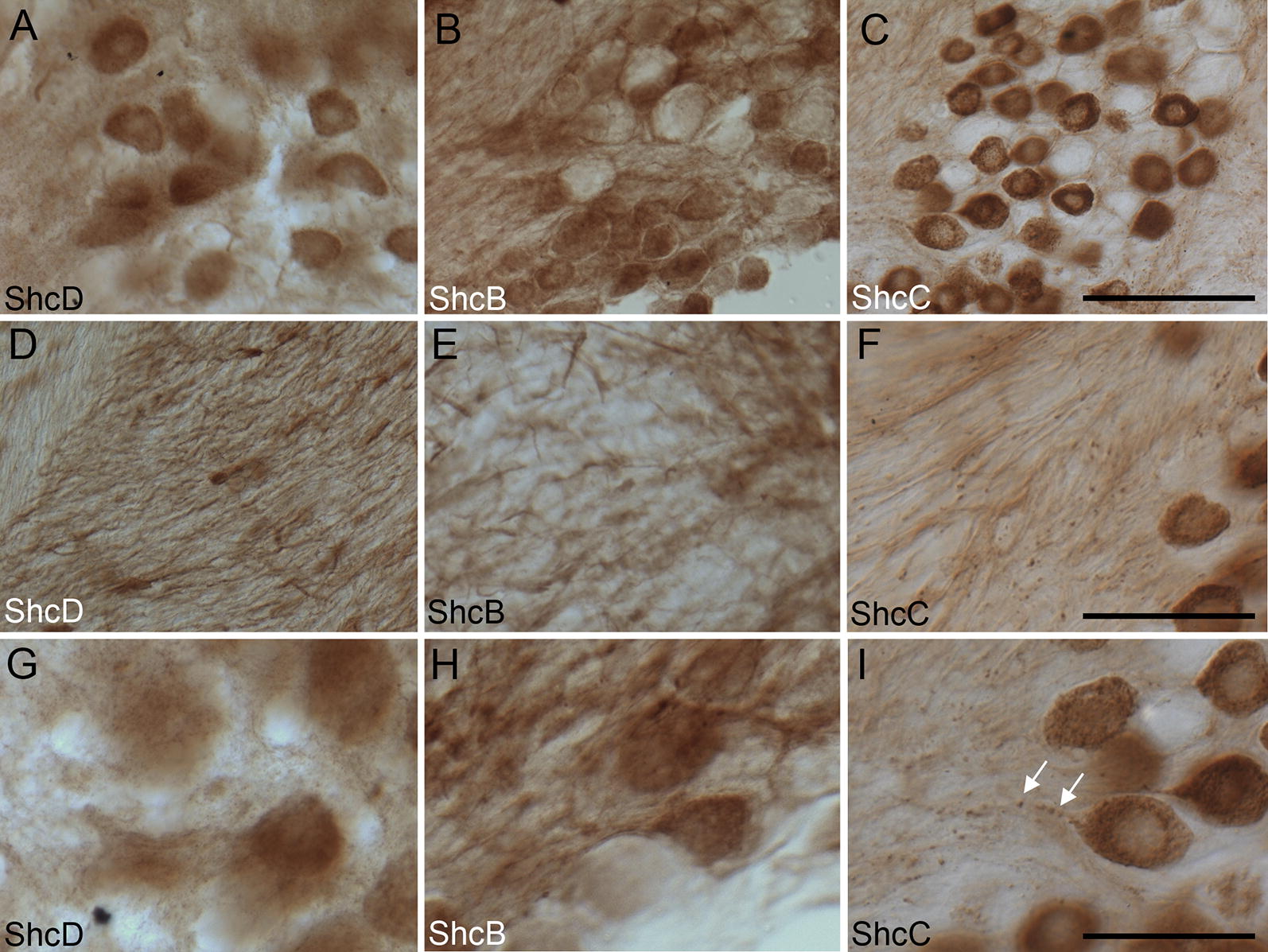
Distribution of ShcD compared with ShcB and ShcC in the rat trigeminal ganglion. Subsets of trigeminal ganglion neurons show strong immunoreactivity in somata and axons for ShcD (**A**, **D**), ShcB (**B**, **E**) or ShcC (**C**, **F**). Under higher power magnification, staining within the axons of these neurons appears either diffuse—ShcD (**G**), ShcB (**H**) or punctate (ShcC (**I**) arrows). Scale bars: **A**–**C** = 100 μm, **D**–**F** = 50 μm, **G**–**I** = 30 μm

In the trigeminal ganglion, prominent staining for all Shc proteins can be observed within the cell bodies of sensory neuron clusters (Fig. 7A–C) as well as in axons (Fig. 7D–F), although the labeling patterns are quite variable. Notably, subpopulations of cells within the ganglion are immunonegative for ShcB and ShcC, and a distinctive ring devoid of immunoreactivity was observed surrounding ShcB and C positive cells, but not around ShcD positive cells (Fig. 7B, C). Furthermore, ShcC appears to localize within discrete puncta which are particularly evident along the axon (Fig. 7I), while ShcB and ShcD remain diffuse (Fig. 7G, H).

### Localization of ShcD within neurons and neural progenitor cells

**Fig. 8:**
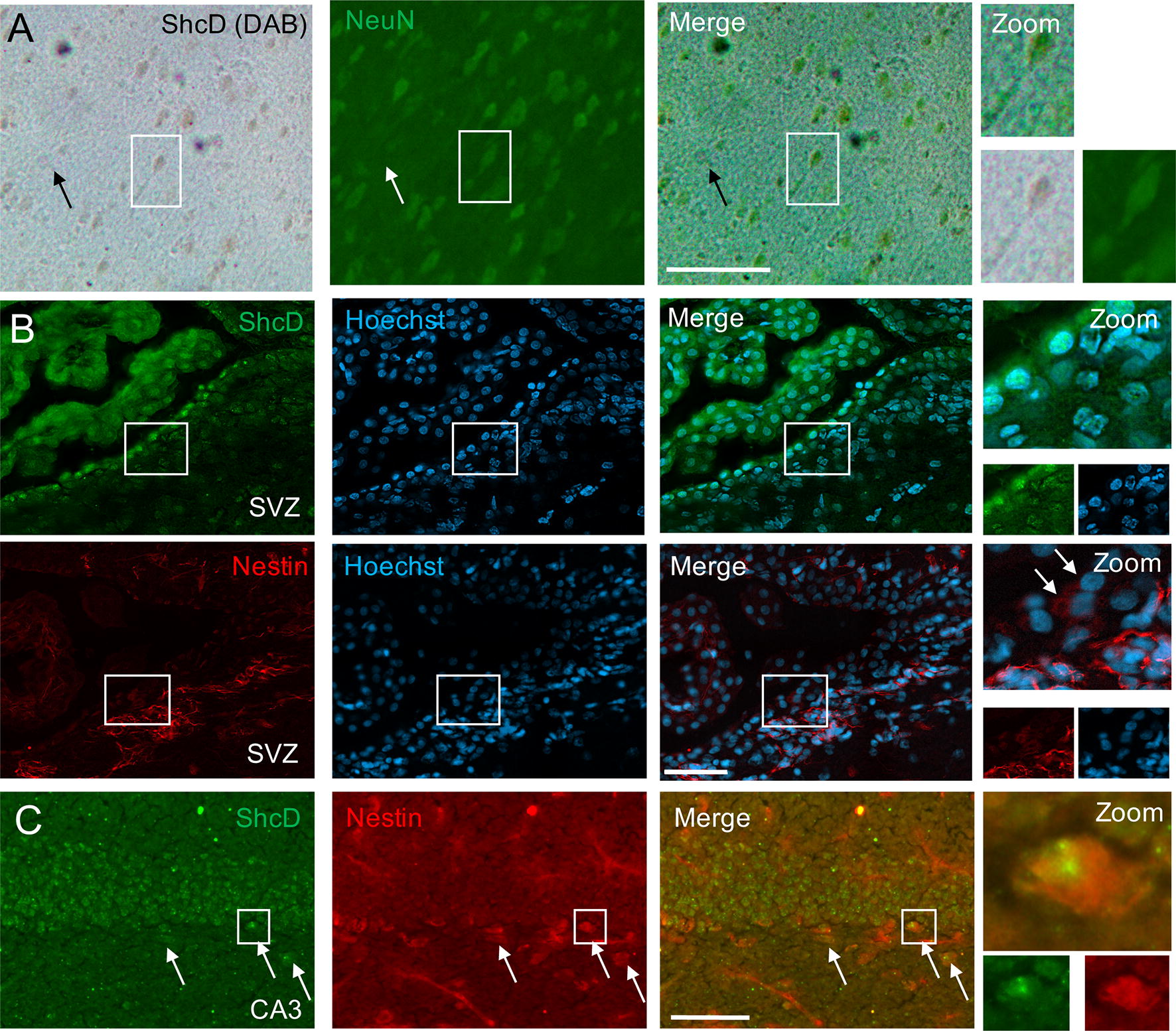
Immunolocalization of ShcD in rat neurons and neural precursors. **A** Dual immunoperoxidase (DAB) and immunofluorescence histochemistry of ShcD and neuronal marker NeuN within the cortex indicates ShcD expression in neurons (see zoom). **B** Immunofluorescence of the subventricular zone (SVZ) on near adjacent coronal sections shows overlapping expression patterns of ShcD and neural precursor marker Nestin, with ShcD staining most prominent in cells lining the lateral ventricle. Hoechst staining denotes nuclei. Arrows indicate nestin expressing cells lining the lateral ventricle. **C** Dual immunofluorescence labeling of the dentate gyrus subgranular layer shows co-staining of ShcD and Nestin (see arrows and zoom). Scale bars: **A**, **C** = 100 μm, **B** = 50 μm

Finally, to precisely determine the identity of cells in the adult nervous system which express ShcD, we performed double staining of ShcD with cell type-specific markers. Dual staining of ShcD with neuronal marker NeuN in the cortex revealed that ShcD is present in mature neurons (Fig. 8A). Additionally, expression of ShcD in neural progenitors was assessed using NPC marker Nestin. Near adjacent coronal sections of the SVZ stained for ShcD or Nestin demonstrate overlapping fluorescent localization patterns (Fig. 8B), and prominent ShcD expression in cells lining the lateral ventricle. Co-staining of ShcD with Nestin in the SGZ of the dentate gyrus supports more directly the possibility of ShcD expression in NPCs (Fig. 8C).

## Discussion

The present immunohistochemical study confirms the widespread presence of ShcB and ShcC in adult neurons, and outlines anatomic and cell type-specific ShcD distribution within the adult CNS for the first time. These expression profiles are summarized in Table 1. In some neuronal populations, all three Shc proteins coexist, while others show restricted expression of only one or two Shc proteins. Cytoplasmic localization was most commonly observed for all Shc proteins. Intriguingly, we uncovered distinct differences in the regional distribution of ShcB, ShcC and ShcD, discussed below, which highlights the complexity of these important neural adaptor proteins.

**Table 1:** Summary of Shc expression patterns in the rat central nervous system

	ShcB	ShcC	ShcD
Olfactory bulb			
Olfactory nerve layer	–	+	+++
Glomerular layer	–	+	+++
Periglomerular interneurons	–	++	+
Tufted cells	–	+	++
Mitral cells	–	++	+
Granule cells	–	++	+
Neocortex and hippocampus			
Neocortex			
Pyramidal cells	+	+++	++
Hippocampus			
CA3	+	+++	++
CA1	+	+++	++
Stratum radiatum	–	+++	++
Dentate gyrus			
Granule cells	+	+++	++
Subgranular zone	+	++	+++
Hilus	+	+++	++
Cerebellum			
Granule cells	–	+++	++
Purkinje cells	++	+++	++
Stellate cells of the molecular layer	–	++	+
Brainstem			
Neuronal somata and dendrites	++	+++	++
Axons and fiber tracts	–	++	–
Dorsal motor nucleus of vagus nerve		+++	+
Hypoglossal nuclei	++	+++	++
Hypoglossal nerve	–	–	–
Spinal trigeminal nerve	–	–	–
Spinal nucleus of the trigeminal nerve	+	+++	++
Spinal cord			
Grey matter			
Dorsal horn	(++)^a^	(+)^a^	++
Ventral horn	(++)^a^	(++)^a^	++
Ventral motor neurons	+++	+++	++
Ventral motor nerves	++	+++	+
White matter			
Axons			++
Trigeminal ganglion			
Neuronal somata	++	+++	++
Axons	++	++	++

Expression levels were attributed as follows: – no expression; + low expression; ++ moderate expression; +++ high expression

^a^As per Ponti et al. [11]

### ShcB, ShcC and ShcD show overlapping expression in motor and sensory neurons

Most of the examined regions showed overlapping expression of all three Shc proteins. Along with ShcD, ShcB and ShcC were present in the adult brainstem and spinal cord, consistent with previous reports [11], and intense staining for all proteins was particularly evident in motor neurons. The nerves in the brainstem show minimal staining for all three neural Shc proteins, while the motor nerves of the spinal cord showed little to no ShcD staining and much less intense ShcB staining. Despite their prominent expression, there have been no reported functions for Shc proteins in motor neurons, though expression of ShcC has been shown to decline upon nerve injury [14]. Similarly, all Shc proteins were found within cell bodies and axons of subsets of sensory neurons in the trigeminal ganglion. ShcB and ShcC are reportedly excluded from supporting glial cells in sensory ganglia [11, 12], and although we were unable to specifically discern glial cells in this study, ShcD transcripts are readily detected in a subtype of multipotent glial cells known as oligodendrocyte precursor cells [15]. Interestingly, ShcC staining in sensory neurons was enriched in puncta around the nucleus and along axons, as reported previously in other neuron subtypes [12]. This punctate pattern might reflect the presence of signaling endosomes that perpetuate Trk signaling from the axon to the cell body through retrograde transport [16]. Indeed, ShcC can associate with Trk RTKs [17, 18], and other Shc molecules have been associated with regulation of RTK endocytosis [9, 19]. Functionally, ShcB and ShcC have been implicated in survival of subpopulations of post-mitotic neurons in other ganglia of the mammalian nervous system, though no overt phenotypes were reported [20]. Notably, ShcD is highly expressed during embryogenesis in both dorsal root and trigeminal ganglia [13], thus it is possible that ShcD could compensate for loss of ShcB and/or ShcC in these and other structures.

### ShcC and ShcD predominate in large projection neurons and interneurons

Neural Shc protein expression was particularly evident in large projection neurons in the cerebellum and hippocampus, although the staining patterns differed for the three proteins. In the cerebellum, all Shc proteins were present in Purkinje cells, ranging in intensity from ShcC (highest), to ShcB, then ShcD (lowest). ShcB staining was restricted to this cell layer, while ShcC and ShcD were also found in the granule cell and molecular layers. The functional role for Shc proteins in this area of the nervous system remains to be determined. Similarly, in the hippocampus, all Shc proteins were present in pyramidal neurons, as well as in smaller-sized neurons, although ShcB was overall barely detectable compared to ShcC and ShcD. ShcC was highly enriched in granule cells of the dentate gyrus, consistent with its involvement in hippocampal long-term potentiation [21]. By contrast, ShcD staining was markedly intense in cells dispersed throughout the SGZ and hilus of the dentate gyrus, including Nestin-positive NPCs. The dentate gyrus is one of three neurogenic niches in the adult brain, and the SGZ represents the source of granule cell precursors [22]. NPCs differentiate in the SGZ, then migrate and integrate into the granule cell layer [23, 24]. Although the physiological function of ShcD is presently unknown, ShcD is upregulated in the early phase of neural differentiation of embryonic stem cells in vitro [25], and it can also promote migration of melanoma cells [7]. It will thus be of future interest to determine whether ShcD influences cellular processes linked to neurogenesis.

### ShcD is uniquely expressed throughout the olfactory bulb

The most overt differences in Shc protein expression occur in the olfactory bulb. Here, intense ShcD staining was observed in the glomerular and olfactory nerve layers, as well as in projection neurons (mitral cells and tufted cells) and interneurons (granule cells). We were unable to confirm ShcD expression in periglomerular interneurons due to the intense overall ShcD staining in glomeruli. Consistent with previous reports [11], ShcB was virtually undetectable across the olfactory bulb, while ShcC was found in mitral cells, granule cells, and periglomerular cells. The olfactory bulb is responsible for processing olfactory inputs, and it also represents a region of new neuron incorporation as the terminal site for NPCs born in the SVZ and olfactory epithelium (OE) neurogenic niches [26–29]. Consistent with their expression patterns, Shc proteins have been previously implicated in aspects of NPC signaling. ShcC is broadly detected in interneurons, where it promotes survival and maturation of neural progenitors [12], while ShcA selectively controls NPC proliferation in the adult SVZ [30]. These opposing roles for ShcA and ShcC in neurogenesis may reflect differences in their signaling properties, including the reduced ability of ShcC to activate Erk compared to ShcA [31]. Intriguingly, ShcD is expressed similarly to ShcA in the SVZ, though ShcD demonstrates additional localization to cells lining the lateral ventricle. In the olfactory system, ShcD is found in an overlapping pattern with ShcC in the main olfactory bulb, and uniquely in the OE and glomeruli, and it has a distinct function in suppressing Erk activation downstream of TrkB [32]. Given the established role of TrkB in migration [33] and differentiation [34] of NPCs, along with its high expression in OE-derived NPCs [35], it is tempting to speculate that ShcD could play a novel role in growth factor-mediated signaling in NPCs.

## Conclusions

In closing, the Shc family has evolved from a single locus in less complex organisms to four distinct loci (ShcA-D) in mammals [1, 36]. While these related adaptors can exhibit compensatory actions, our findings extend the notion that differences in expression patterns and signaling properties likely promote non-redundant functions of Shc proteins within the adult mammalian CNS.

## Methods

### Animals

Adult male Sprague–Dawley rats (200–300 g; Charles River, QC) were housed under standard conditions with free access to food and water. A total of ten rats were used in this study; 5 rats were used for IHC, three rats were used for IF, and two rats were used in antibody control experiments. 8 weeks old rats were used in all figures, except for Fig. 8 where 12–14 weeks old rats were used. Animals were euthanized by intraperitoneal sodium pentobarbital injection (120 mg/kg) or CO_2_ inhalation (plexiglass chamber with a flow rate of 6.0 L/min (30% air volume) for at least 5 min) followed by decapitation. All procedures were carried out in accordance with the guidelines established by the Canadian Council on Animal Care, and approved by the University of Guelph Animal Care Committee (Guelph, ON) under animal utilization protocol #06006R.

### Antibodies

The following primary antibodies were obtained commercially and used at the indicated dilutions: mouse anti-Nestin (1:200, R&D Systems, Minneapolis, MN; MAB2736), mouse anti-NeuN (1:500; Millipore, Etobicoke, ON; MAB377), rabbit anti-ShcB (1:1000; Santa Cruz, CA; sc-33,808). Rabbit anti-ShcC (1:2000) was kindly provided by Dr. John O’Bryan (Medical University of South Carolina, Charleston, SC). Rabbit anti-ShcD (1:1000) was generated against the unique CH2 region. Its specificity has been confirmed by preadsorption with the original antigen prior to immunohistochemistry [8, 13], by immunoblot against lysates prepared from cells expressing other Shc proteins [8], and by immunoblot on brain lysates prepared from ShcD knockout mice [32].

### Immunoperoxidase histochemistry

Animals were deeply anaesthetized with sodium pentobarbital (120 mg/kg) and slowly perfused through the ascending aorta with 60 mL of ice cold, artificial cerebrospinal fluid (85 mM NaCl, 2.5 mM KCl, 1.25 mM sodium phosphate monobasic, 25 mM NaHCO_3_, 25 mM glucose, 75 mM sucrose, 0.5 mM CaCl_2_, 4 mM MgCl_2_, 100 µM kynurenic acid), saturated with 95% O_2_/5% CO_2_. This solution has been shown previously to aid in preserving the integrity of cell membranes in the CNS (adapted from [37]). Following washout of the blood, animals were slowly perfused with 60 mL of an ice cold solution containing 2% paraformaldehyde (PFA) in 100 mM phosphate buffer (pH 6.5), followed by 120 mL of 2% PFA in 100 mM phosphate buffer (pH 8.5) (adapted from the method described by [38]). The brains and spinal cords were then removed from the skull and post-fixed overnight in 2% PFA in 100 mM phosphate buffer (pH 8.5) at 4 °C, sectioned in the coronal or parasagittal plane at 50 μm on a vibrating microtome (Vibratome 3000 Plus) and stored in Millonig’s buffer (pH 7.4) containing 0.04% sodium azide. Sections were processed according to the method described by [38]. Briefly, free-floating sections were placed into 6-well culture plates (5 mL per well; Corning) on a slowly rotating, orbital shaker, washed for 30 min in 1% H_2_O_2_ in 25 mM Tris-buffered saline (TBS), and incubated in 0.2% Triton X-100. Sections were next rinsed for 5 min in TBS and 2× 15 min in TBS containing 0.005% IgG-free BSA (Jackson ImmunoResearch, West Grove, PA). Sections were then blocked for 3 h in TBS + 0.005% BSA + 5% normal goat serum and reacted overnight in the same solution with indicated antibodies. The next day, sections were washed 2 × 15 min in TBS, reacted for 1 h in TBS containing biotinylated secondary antibodies (1:1000; Jackson ImmunoResearch, West Grove, PA), washed 2 × 15 min in TBS, reacted again for 1 h in TBS containing peroxidase-conjugated avidin–biotin complex (1:1000, ABC™ Elite, Vector Labs, Burlington, ON, Canada), washed 3 × 5 min in TBS and reacted for 15 min in TBS containing diaminobenzidine (DAB: Sigma, St. Louis, MO;#4293). Sections were attached to slides and allowed to dry overnight, then dehydrated in increasingly concentrated ethanol solutions before mounting in xylene. All washes and reactions took place at room temperature. Controls included omission of primary antibodies on adjacent sections. Images were captured using an AxioCam MRc5 digital camera (Carl Zeiss Canada). Images were post-processed for brightness and color balance and assembled in Adobe Photoshop 10.0 (San Jose, CA).

### Immunofluorescence histochemistry

Rats were euthanized by CO_2_ inhalation followed by decapitation. Brains were removed from the skull, fresh-frozen in 2-methylbutane (Fisher Scientific, Pittsburgh, PA; O3551-4), cooled with dry ice and stored at − 80 °C. Samples were then embedded in Cryomatrix (ThermoFisher Scientific, Waltham, MA; #6769006) and sectioned directly onto Superfrost Plus (Fisher Scientific; #12-550-15) slides at 20 μm using a cryostat (Leica CM1860). Slides were warmed briefly in a 37 °C incubator prior to staining. All steps were performed in a humidified chamber. Slides were fixed using 4% PFA in phosphate buffered saline (PBS), washed with PBS, permeabilized with 0.1% Triton X-100 in PBS, and blocked using 10% normal goat serum, 1% BSA and 0.1% Triton X-100 in PBS. Primary antibodies for ShcD (1:150 CH2) and Nestin were diluted in blocking buffer and incubated overnight at 4 °C either separately or together. Slides were then incubated with appropriate secondary antibodies for 30 min with 0.2% Triton X-100 in PBS, next in Hoechst 33258 (1:2000; Molecular Probes, Eugene OR; H3569) for 2 min, and then coverslipped using ProLong™ Diamond Antifade Mountant (ThermoFisher Scientific, Waltham, MA; P36965). In the case of dual immunoperoxidase/fluorescence histochemistry, slides were incubated with 3% H_2_O_2_ in PBS in place of permeabilization. Primary antibody for ShcD (1:1000 CH2) was diluted in blocking buffer and incubated overnight at 4 °C. Biotinylated goat anti-rabbit secondary antibody (1:500), diluted in 0.2% Triton X-100 in PBS, was added for 1 h at room temperature. Next, sections were incubated with PBS containing peroxidase-conjugated avidin-biotin complex (1:1000; ABC™ Elite, Vector Labs, Burlingame, CA; PK-6100) and 0.2% Triton X-100. Slides were then treated with 0.1 mg/mL 3,3′-diaminobenzidine solution (DAB: Polysciences Inc. Warrington, PA; 04001-5) in PBS until colour change was observed (10–20 min). From here, immunofluorescence protocol was performed as indicated above beginning with primary antibody for NeuN. Images were captured on a Leica DMIRE2 fluorescent microscope, Leica DM1000 with OMAX A3580U color camera or Nikon Ti2E fluorescent microscope and assembled in Adobe Photoshop 12.0 (San Jose, CA).

## Data Availability

The data and materials are available from the corresponding author upon request.

## Abbreviations

ac: anterior commissure
aca: anterior limb of the anterior commissure
CH1: collagen homology 1
CH2: collagen homology 2
CNS: central nervous system
cst: corticospinal tract
ctx: cortex
dmv: dorsal motor nucleus of the vagus nerve
epl: external plexiform layer
gcl: granule cell layer
glm: glomerular layer
hip: hippocampus
ica: intrabulbar limb of the anterior commissure
icp: inferior cerebellar peduncle
ipl: inner plexiform layer
lfp: longitudinal fasciculus of the pons
mcl: mitral cell layer
med: cerebellum
mol: molecular layer
NPC: neural precursor cell
ob: olfactory bulb
onl: olfactory nerve layer
Pc: Purkinje cell
PTB: phosphotyrosine binding
RaLP: Rai-like protein
RTK: receptor tyrosine kinase
Scp: superior cerebellar peduncle
sgz: subgranular zone
Shc: Src homology and collagen
spV: spinal nucleus of the trigeminal nerve
spVn: spinal trigeminal nerve
SVZ: subventricular zone
XII: hypoglossal nuclei
XIIn: hypoglossal nerve
